# Diagnostic accuracy of the visceral adiposity index in patients with metabolic-associated fatty liver disease: a meta-analysis

**DOI:** 10.1186/s12944-022-01636-8

**Published:** 2022-03-06

**Authors:** Xianhao Yi, Shaihong Zhu, Liyong Zhu

**Affiliations:** Department of General Surgery, The Third Xiangya Hospital, Central South University, No.138, Tongzipo Road, Yuelu District, Hunan 410013 Changsha, China

**Keywords:** Diagnostic accuracy, Visceral adiposity index, Metabolic-associated fatty liver disease, Meta-analysis

## Abstract

**Background:**

Conflicting results on the prognostic value of the visceral adiposity index (VAI) in patients with metabolic-associated fatty liver disease (MAFLD) have been reported. This study aimed to assess the diagnostic value of the VAI in MAFLD patients.

**Methods:**

The Cochrane Library, PubMed, Embase, and other databases were searched to collect all documents that met the inclusion criteria from the establishment of the database to September 2021. The methodological quality of the included studies was assessed using the Newcastle–Ottawa Scale. The heterogeneity among the studies was analysed by the Cochran Q test and I^2^ test, and the appropriate model was selected according to the heterogeneity results. The diagnostic efficacy of the VAI was evaluated by sensitivity, specificity, and area under the curve, and a Fagan diagram was generated to evaluate the diagnostic ability of the VAI.

**Results:**

A total of 9 studies were included. The overall quality of the included studies was good. Meta-analysis showed that the combined sensitivity of the VAI for the diagnosis of MAFLD was 0.70 [95% CI (0.69–0.71)], the combined specificity was 0.67 [95% CI (0.67–0.68)], the combined positive likelihood ratio was 2.08 [95% CI (1.87–2.31)], the combined negative likelihood ratio was 0.39 [95% CI (0.34–0.44)], and the combined diagnostic odds ratio was 5.81 [95% CI (4.73–7.14)]. The corresponding area under the curve was 0.79 [95% CI (0.75–0.82)]. Meta-regression analysis showed that the diagnostic method was a potential source of heterogeneity (*P* < 0.05). The Fagan diagram showed that the precision of MAFLD diagnosis was 70% when the pretest probability was set to 50% and then supplemented by the VAI.

**Conclusions:**

The VAI is an independent predictor in the diagnosis of MAFLD and may be helpful in the detection of MAFLD. A VAI > 2.33 suggests that patients have a high probability of having MAFLD.

**Supplementary Information:**

The online version contains supplementary material available at 10.1186/s12944-022-01636-8.

## Background

Metabolic-associated fatty liver disease (MAFLD) is an increasingly common disease, with a prevalence of 25.24% worldwide [1]. The detection of MAFLD is of great importance for its treatment and prognosis, and liver biopsy is the gold standard for the diagnosis of MAFLD, but its invasive nature limits its clinical application. The noninvasive diagnostic methods include magnetic resonance imaging, computed tomography, the MAFLD fibrosis score, and the Fibrosis-4 index, but these methods have disadvantages such as high costs and complicated indicators [2]. The main pathological manifestation of MAFLD is fat infiltration in the liver tissue. Studies have shown that the fatty liver index, lipid accumulation products, and the hepatic steatosis index have clinical significance for the diagnosis and evaluation of MAFLD [3]. These indicators consist of simple anthropometric measurements and laboratory tests and represent the state of lipid metabolism in the body. Studies have reported that visceral fat, as a representation of the total fat in the abdominal cavity, is closely related to fat infiltration in the liver tissue [4, 5]. The visceral adiposity index (VAI) is a new scoring system based on waist circumference, body mass index, triglycerides, and high-density lipoprotein. The VAI is an important index that was recently proposed to assess the distribution and dysfunction of visceral fat and is closely related to cardiovascular and cerebrovascular risk [6]. At present, there is still controversy about whether the VAI can accurately diagnose MAFLD. Vongsuvanh et al. [7] reported that the VAI is not a predictor of liver histology in patients with MAFLD. However, many scholars believe that the VAI is a practical tool in patients with biopsy-proven MAFLD [8, 9]. This study systematically evaluated whether the VAI can accurately diagnose MAFLD through meta-analysis, aiming to provide reliable evidence as a foundation for clinical research.

## Methods

### Literature search

This review was conducted following the preferred reporting items for systematic reviews and meta-analysis (PRISMA) guidelines [10] and has been registered in the International Prospective Register of Systematic Reviews (registration number: CRD42021275084). A complete and computerized search of the Cochrane Library, PubMed, and Embase was performed independently by two authors, without restrictions based on region, language, or publication type. Clinical studies related to the diagnosis of MAFLD with the VAI were searched from database establishment to September 2021. Keywords were combined with free words for retrieval. The English search terms included nonalcoholic steatohepatitis, nonalcoholic fatty liver disease, metabolic-associated fatty liver disease, NASH, NAFLD, MAFLD, visceral adiposity index, and VAI. The detailed search strategy is shown in Additional file 1. We also searched for grey literature using Google.

### Study selection

Two authors (XH Y and LY Z) independently screened the literature, extracted data, and evaluated the methodological quality of the included studies according to the inclusion and exclusion criteria. In cases of disagreement, the dispute was settled through discussion or adjudicated by a third investigator (SH Z).

The inclusion criteria were as follows: (1) the paper was a primary or original study, including a case–control studies and randomized controlled trials; (2) the paper diagnosed MAFLD by imaging examination or biopsy; (3) the paper had a control group with a comparable age; (4) the paper compared the VAI between the MAFLD group and the control group; and (5) the paper provided sufficient and available data (the true-positive value (TP), false-positive value (FP), false-negative value (FN), and true-negative value (TN) were provided or could be calculated statistically [8] [11]).

The exclusion criteria were as follows: (1) conference reports; (2) reports that were missing important data or had unclear measurement indicators; and (3) reports published repeatedly in different journals (when two studies from the same institution reported the same target outcome, the quality of inclusion was better); and (4) studies whose titles were related to the VAI-based diagnosis of MAFLD but whose content was not related to the VAI-based diagnosis of MAFLD.

### Data extraction

Two independent investigators (XH Y and LY Z) reviewed the full-text articles. Interresearcher disagreements were resolved by a third investigator (SH Z). A data extraction table was developed to extract data, and the table included the following: (1) first author, year of publication, and country; (2) sample size, TP, FP, FN, TN, sensitivity (Sen), specificity (Spe), positive likelihood ratio (PLR), negative likelihood ratio (NLR), diagnostic odds ratio (DOR), subject operating characteristic curve (SROC) and area under the curve (AUC), and quality evaluation of the literature; (3) methods for diagnosing MAFLD; and (4) methods for detecting and calculating the VAI and the value of the VAI. The outcome was measured as the mean ± SD or MD with 95% confidence intervals (CIs). If there were discrepancies between reviewers, a joint reevaluation of the original article was performed.

### Risk of bias assessment

The methodological quality of the included studies was assessed independently by two authors (XH Y and LY Z) using the Newcastle–Ottawa Scale (NOS) [12]. According to this tool, study quality is assessed based on eight items that are categorized into three groups: study group selection, group comparability, which bases its assessment on the selection of the study groups and the comparability of study groups, and ascertainment of the outcome. Studies awarded 7 or more stars were deemed high quality.

### Data synthesis and analysis

RevMan 5.3 and Meta-Disc 1.4 software were used for analysis. Spearman correlation analysis was used to investigate whether there was a threshold effect, and the SROC curve was summarized to determine whether the SROC pattern exhibited a “shoulder and arm shape”. The Cochran Q test and I^2^ test were used to analyse the heterogeneity among the included studies. Appropriate models were selected according to the heterogeneity (if *P* > 0.05 and/or I^2^ < 50%, the fixed-effects model was used for meta-analysis; otherwise, a random-effects model was used for meta-analysis). The source of heterogeneity was identified by subgroup analysis and meta-regression analysis. Additionally, subgroup analysis was performed based on diagnostic method, control source, and race. The data were also plotted (SROC) to establish the TP and FP of each study, and the one-by-one exclusion method was used for sensitivity analysis to evaluate the stability of the study results. Stata 16.0 software was used to draw Deek’s funnel plot to evaluate publication bias. When *P* > 0.05, it was assumed that no publication bias was present. A Fagan plot was used to evaluate the diagnostic ability of the VAI. *P* < 0.05 was considered to indicate statistical significance.

## Results

### Study selection

**Fig. 1:**
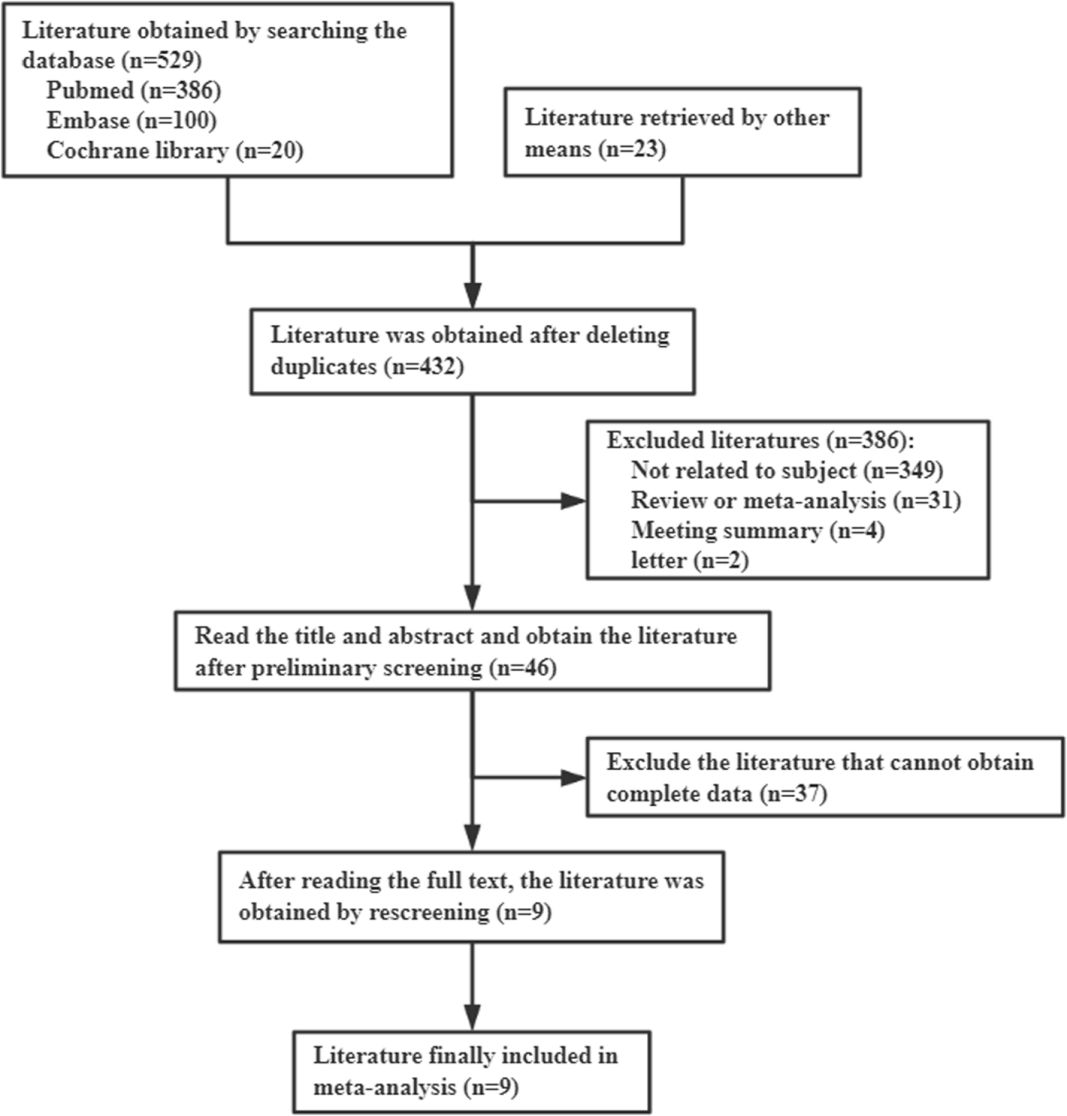
Flow chart of literature screening

A total of 652 articles were initially obtained from the databases and other sources based on keywords (Fig. 1). Among these articles, 220 duplicate articles were removed, and 432 articles remained. By reviewing the titles and abstracts, 46 articles were left for further full-text review. Then, we reviewed the full texts of these articles carefully and excluded an additional 37 articles. Finally, 9 articles [8, 13–20] were included.

### Characteristics of the included studies

The main characteristics of the 9 studies included are listed in Table 1. The included studies included 14,486 cases and 23,702 controls. Three studies were performed in China, one in France, one in America, one in Turkey, one in Italy, one in Mexico, and one in Greece. Two of the 15 included studies adopted liver biopsy to diagnose MAFLD, six studies diagnosed MAFLD by ultrasonography, and one diagnosed MAFLD by computed tomography. Two studies included healthy people, and seven studies included non-MAFLD people as the control group. The cut-off of the VAI varied from 1.25 to 2.33.

**Table 1:** Basic characteristics of included studies

Author	Year	Country	Diagnostic methods	Control source	Race	Cases number	Control number	Cut-off	TP	FP	FN	TN
Cen	2020	China	Ultrasonographic	Non-MAFLD	Asian	6261	10,207	1.59	4414	3205	1847	7002
Fedchuk	2014	France	Biopsy	Non-MAFLD	European	241	83	1.25	190	7	51	76
Fu	2019	America	Computed tomography	Non-MAFLD	European	40	67	1.46	35	42	5	25
Keskinler	2020	Turkey	Biopsy	Healthy	European	57	57	1.78	45	16	12	41
Li	2017	China	Ultrasonographic	Non-MAFLD	Asian	7324	12,480	1.89	4988	4152	2336	8328
Lin	2021	China	Ultrasonographic	Non-MAFLD	Asian	354	410	-	293	192	61	218
Musso	2012	Italy	Ultrasonographic	Healthy	European	41	82	1.9	31	23	10	59
Ortega	2019	Mexico	Ultrasonographic	Non-MAFLD	European	36	158	2.33	28	75	8	83
Vassilatou	2018	Greece	Ultrasonographic	Non-MAFLD	European	132	158	-	85	34	47	124

Rank by the beginning letter of the first authors

Abbreviations: *TP* True positive value, *FP* False positive value, *FN* False negative value, *TN* True negative value, *MAFLD* metabolic-associated fatty liver disease.

### Quality assessment of the included studies

As shown in Table 2, quality assessment of the included studies was carefully performed using the NOS, and the general quality was moderate (four studies scored 8 points, two scored 7 points, and three scored 6 points, mean ± SD: 7.11 ± 0.87), indicating relatively high quality. No study was excluded due to a poor NOS score.

**Table 2:** Quality assessment of included studies using the Newcastle-Ottawa Scale

First author	Selection^a^				Comparability^b^	Exposure^c^			Score
	1	2	3	4		A	B	C	
Cen [16]	☆	☆	-	☆	☆	☆	☆	☆	7
Fedchuk [11]	☆	☆	-	-	☆	☆	☆	☆	6
Fu [14]	☆	☆	☆	☆	☆	☆	☆	☆	8
Keskinler [18]	☆	☆	-	☆	☆☆	☆	☆	☆	8
Li [12]	☆	☆	-	☆	☆☆	☆	☆	☆	8
Lin [17]	☆	☆	☆	☆	☆	☆	☆	☆	8
Musso [10]	☆	☆	-	-	☆	☆	☆	☆	6
Ortega [15]	☆	☆	-	-	☆	☆	☆	☆	6
Vassilatou [13]	☆	☆	-	☆	☆	☆	☆	☆	7

Rank by the beginning letter of the first authors

^a^1: Is the case definition adequate? 2: Representativeness of the cases. 3: Selection of Controls. 4: Definition of Controls

^b^Comparability: Comparability of cases and controls on the basis of the design or analysis. A maximum of two stars can be given for Comparability

^c^A: Ascertainment of exposure. B: Same method of ascertainment for cases and controls. C: Nonresponse rate

### Meta-analysis results

**Fig. 2:**
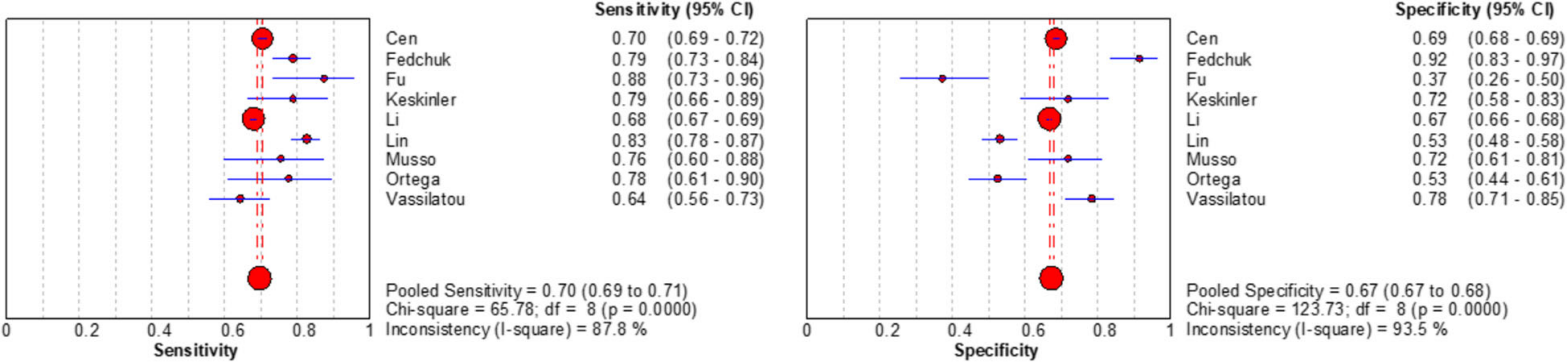
Forest plot assessing the pooled sensitivity and specificity of VAI in the diagnosis of MAFLD in included studies

**Fig. 3:**
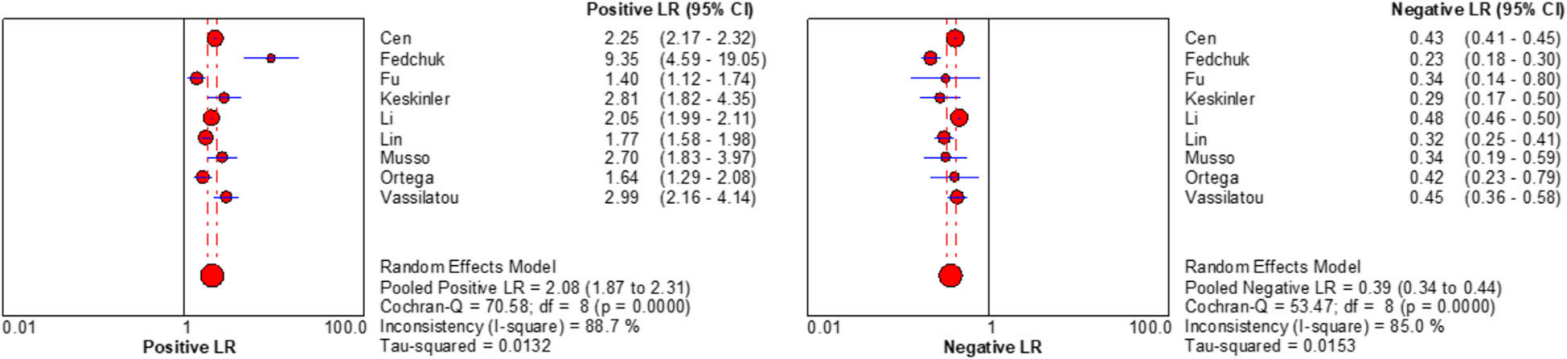
Forest plot assessing the pooled positive and negative likelihood ratios of VAI in the diagnosis of MAFLD in included studies

**Fig. 4:**
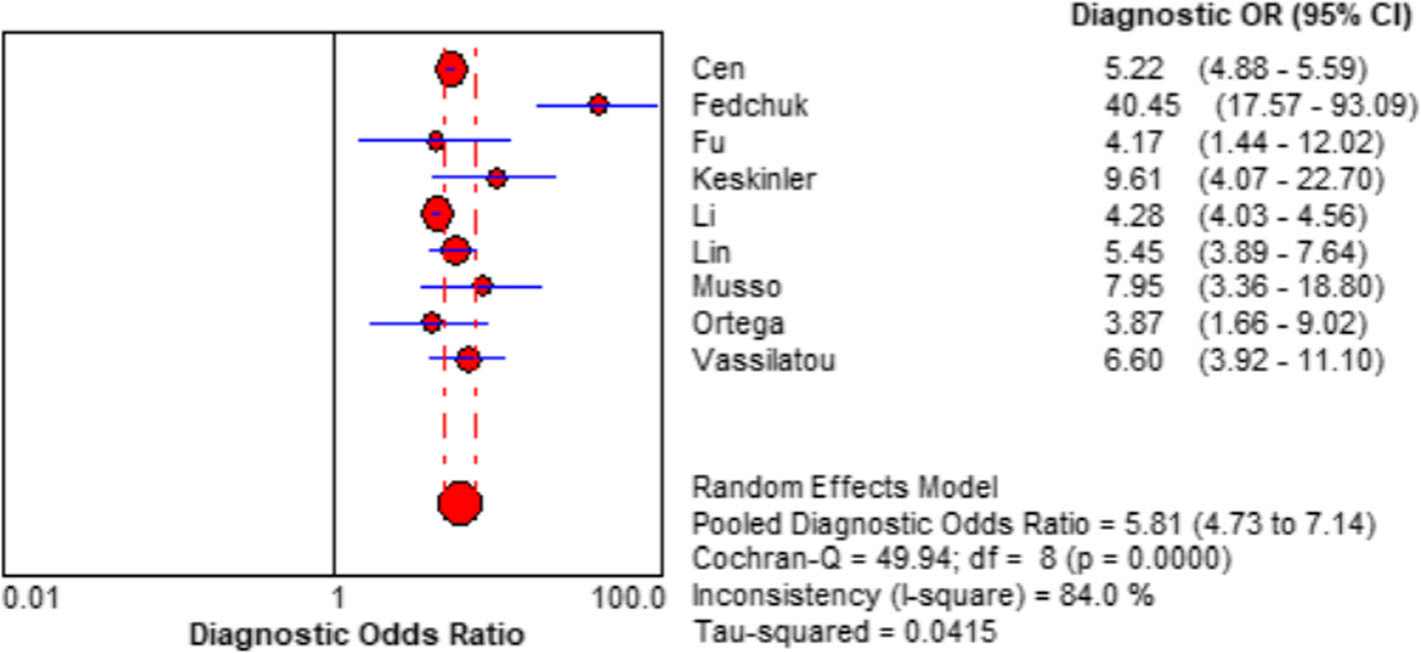
Forest plot assessing the diagnostic odds ratio of VAI in the diagnosis of MAFLD in included studies

**Fig. 5:**
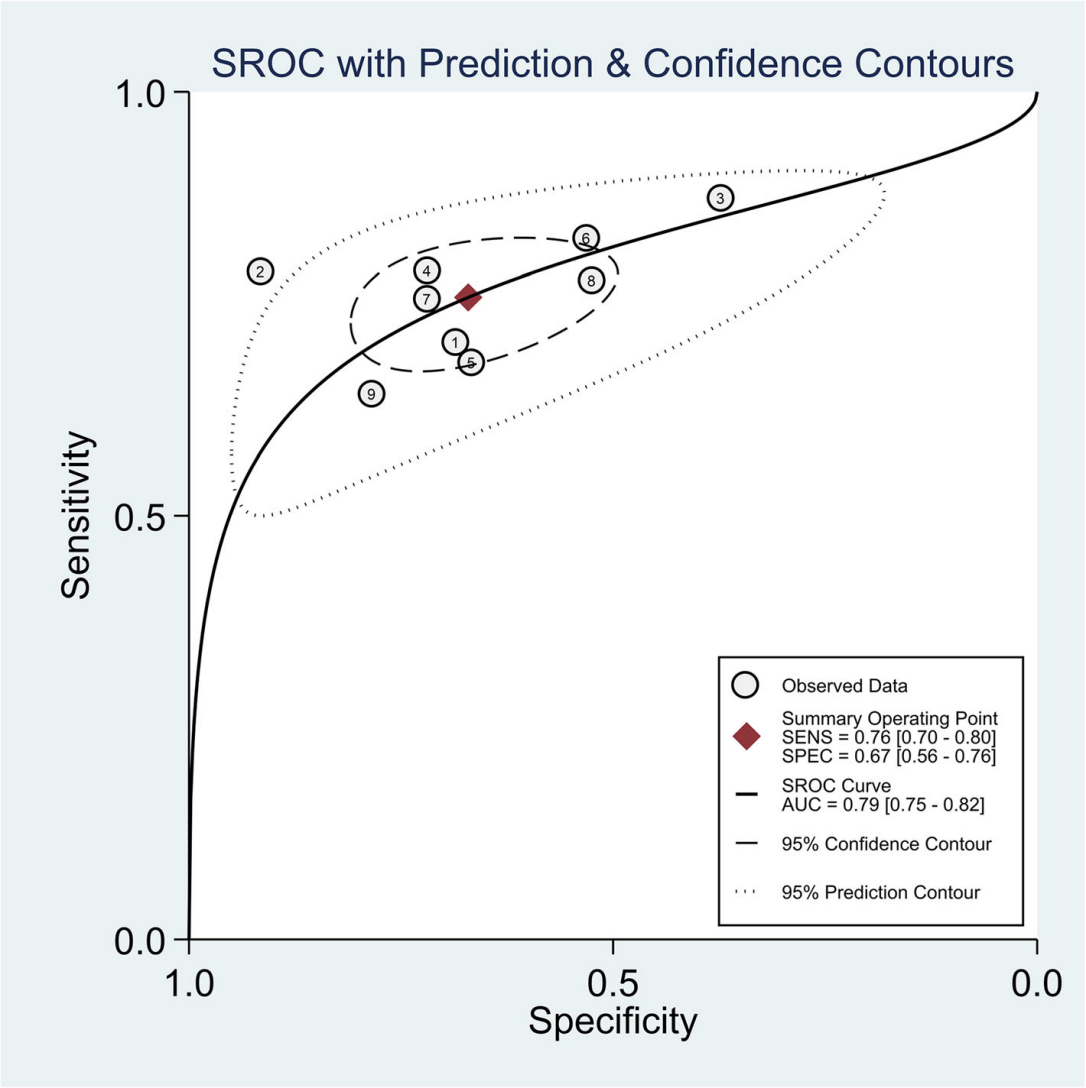
Summary receiver operating curve of the diagnosis performance of VAI for MAFLD in included studies

Spearman rank correlation analysis showed that sensitivity was positively correlated with (1-specificity) (rs = 0.450, *P* = 0.224), and there was no threshold effect. The heterogeneity of the VAI was analysed by Sen, Spe, PLR, NLR, and DOR. The results showed that there was heterogeneity (*P* < 0.001, I^2^ = 87.8%; *P* < 0.001, I^2^ = 93.5%; *P* < 0.001, I^2^ = 88.7%; *P <* 0.001, I^2^ = 85.0%; *P* < 0.001, I^2^ = 84.0%, respectively), and meta-analysis was carried out with a random effect model. The results showed that the combined Sen of the VAI in the diagnosis of MAFLD was 0.70 [95% CI (0.69–0.71)], the combined Spe was 0.67 [95% CI (0.67–0.68)], the combined PLR was 2.08 [95% CI (1.87–2.31)], the combined NLP was 0.39 [95% CI (0.34–0.44)], and the combined DOR was 5.81 [95% CI (4.73–7.14)]. The corresponding AUC was 0.79 [95% CI (0.75–0.82)], as shown in Figs. 2, 3, 4 and 5.

### Subgroup analysis and meta-regression analysis

**Fig. 6:**
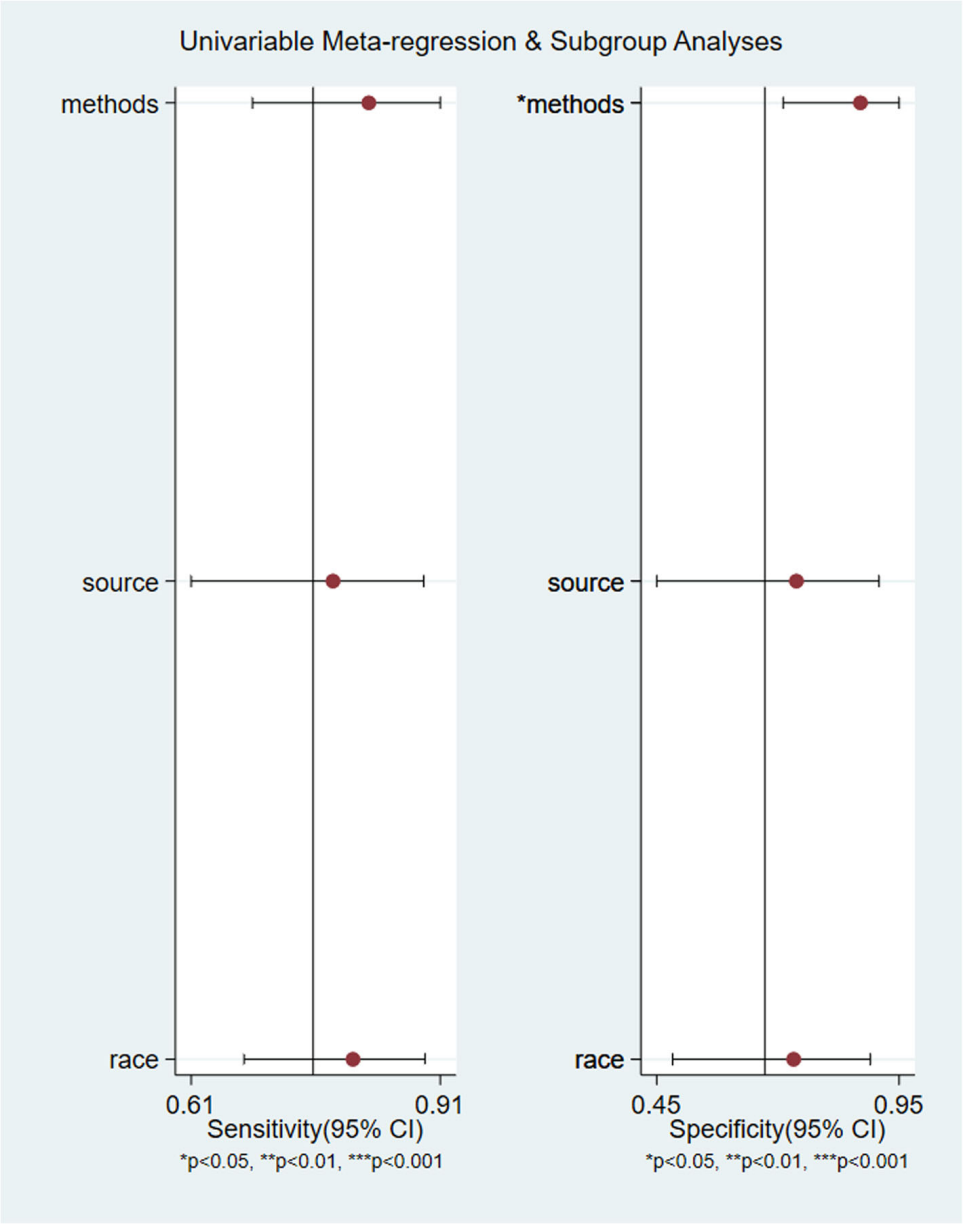
Meta-regression analysis of VAI in the diagnosis of MAFLD in included studies

After subgroup analysis based on three factors, diagnostic method, control source, and race, it was found that the Sen, Spe, PLR, and NLR of VAI in the diagnosis of MAFLD with biopsy as the diagnostic method were better than those with imaging examination as the diagnostic method (Table 3 for details). The DOR of the VAI in the diagnosis of MAFLD with biopsy as the diagnostic method was 19.79 [95% CI (4.8-81.63)], which wassignificantlyhigher than the 4.96[95% CI (4.26–5.79)] obtained for imaging examination (*P*<0.05). Meta-regression analysis also showed that the diagnostic method was a potential source of heterogeneity,*P*<0.05. Control source (*P*=0.76) and race (*P*=0.42) were not heterogeneous (Fig. 6 for details).

**Table 3:** Subgroup and Meta-regression analysis of VAI in the diagnosis of MAFLD in included studies

Subgroup	Number of studies	Sen(95%CI)	Spe(95%CI)	PLR(95%CI)	NLP(95%CI)	DOR(95%CI)	RDOR(95%CI)	*P*
Diagnostic methods								
Biopsy	2[11, 18]	0.79(0.74–0.83)	0.84(0.76–0.89)	5.00(1.27–19.72)	0.24(0.19–0.30)	19.79(4.8-81.63)	4.06(1.87–8.79)	0.0036
Imaging examination	7[10, 12–17]	0.70(0.69–0.70)	0.67(0.67–0.68)	1.99(1.80–2.19)	0.43(0.39–0.47)	4.96(4.26–5.79)		
Control source								
Healthy	2[10, 18]	0.78(0.68–0.85)	0.72(0.64–0.79)	2.75(2.06–3.67)	0.31(0.21–0.46)	8.78(4.76–16.06)	1.38(0.35–5.40)	0.5898
Non-MAFLD	7[11–17]	0.70(0.69–0.70)	0.67(0.67–0.68)	2.01(1.80–2.25)	0.39(0.35–0.45)	5.55(4.48–6.88)		
Race								
Asian	3[12, 16, 17]	0.70(0.69–0.70)	0.67(0.67–0.68)	2.04(1.86–2.24)	0.43(0.39–0.48)	4.84(4.07–5.75)	1.70(0.69–4.22)	0.2091
European	6[10, 11, 13–15, 18]	0.76(0.72–0.79)	0.67(0.64–0.71)	2.66(1.61–4.38)	0.33(0.24–0.45)	8.39(4.38–16.05)		

Abbreviations: *Sen Sensitivity* Spe Specificity, *PLR* Positive likelihood ratio, *NLR* Negative likelihood ratio, *DOR* Diagnostic odds ratio, *RDOR* Relative diagnostic odds ratio, *MAFLD* metabolic-associated fatty liver disease

The *P* value represents the statistical difference of DOR between different groups

### Sensitivity analysis and publication bias

**Fig. 7:**
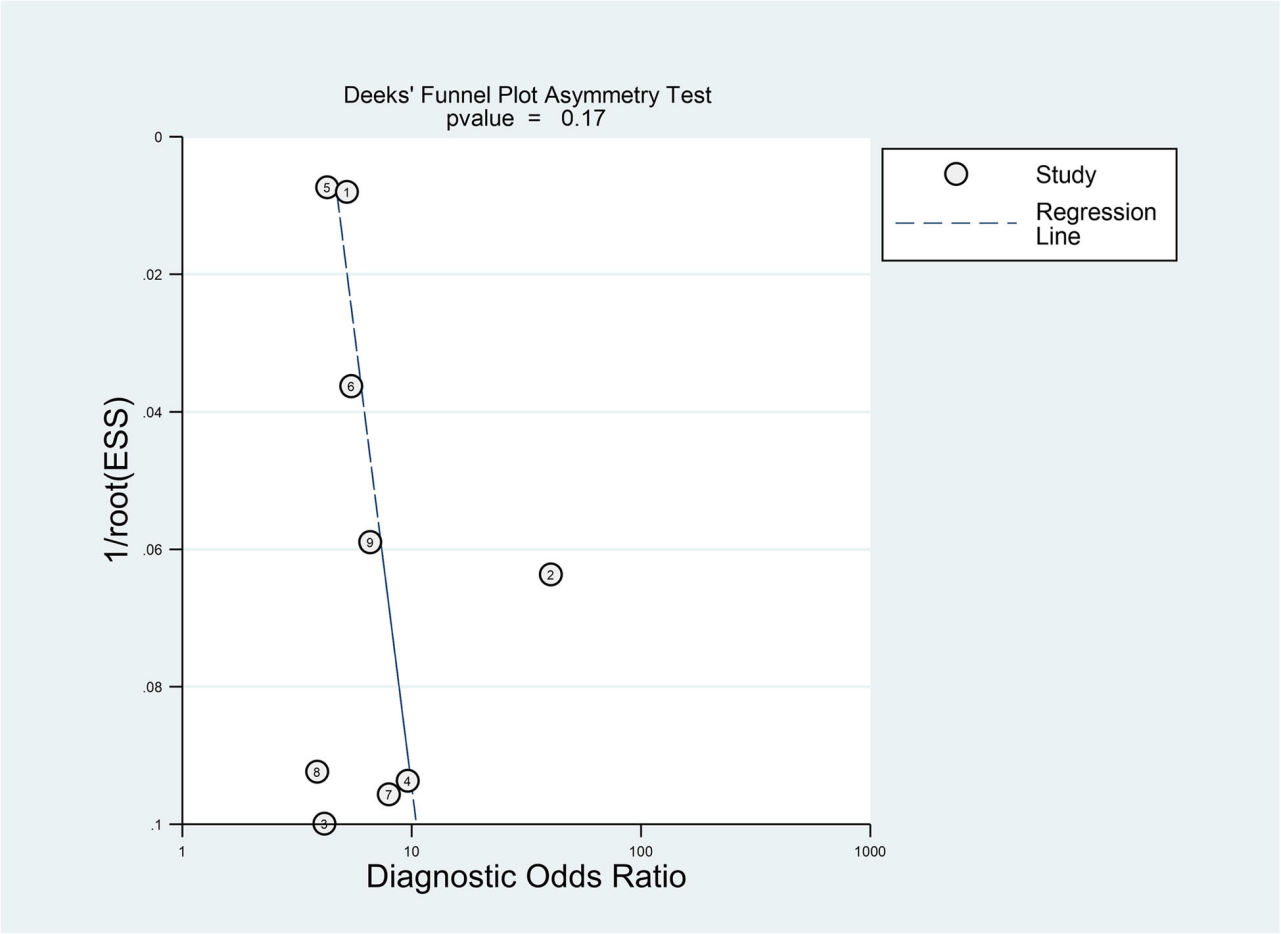
Deek’s funnel plot assessing the publication bias of included studies

The sensitivity analysis was carried out by excluding the included studies one by one. The results showed that the individual included studies had no significant impact on the results. Deek’s funnel plot showed that there was no potential publication bias among the included studies (*P*=0.17) (Fig. 7).

### Clinical application value

**Fig. 8:**
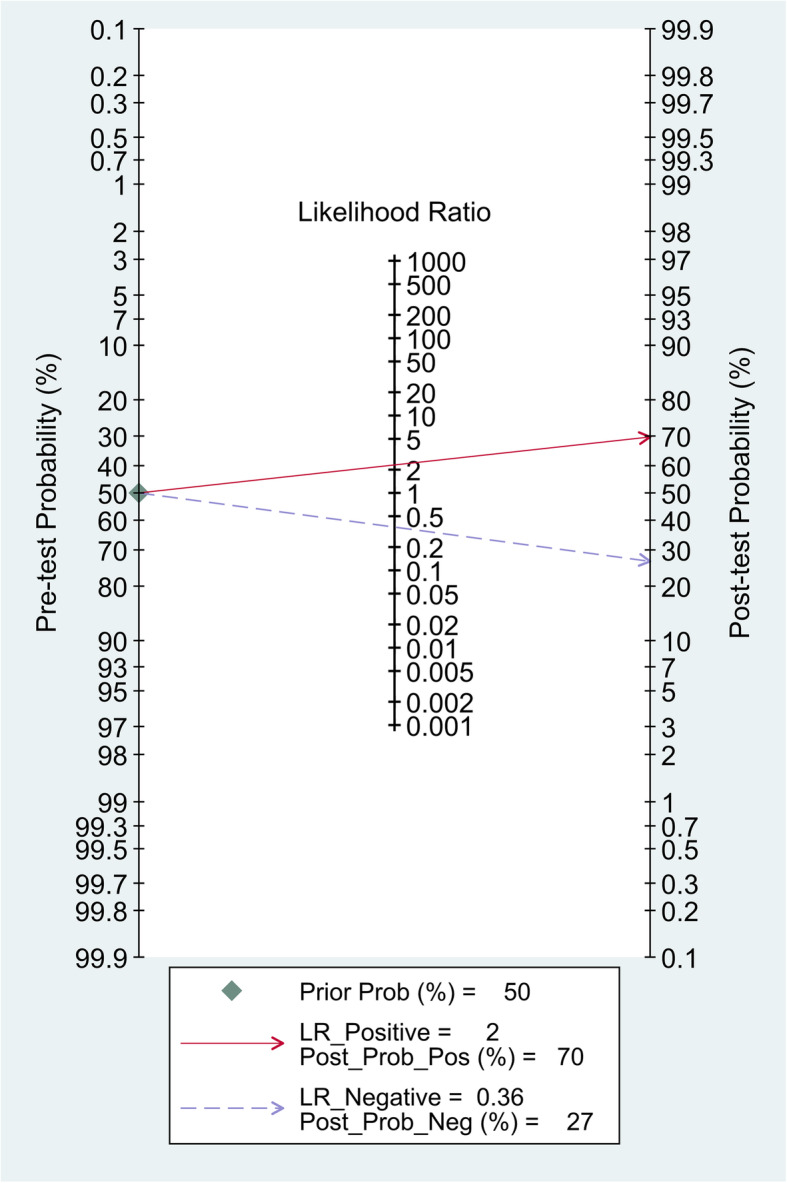
Fagan’s nomogram showing the diagnostic value of VAI for MAFLD

A Fagan diagram was drawn, the pretest probability was set as 50%, and then the VAI was measured. In the included studies, the value of VAI ranged from 1.25 to 2.33. When the VAI was > 2.33, the accuracy of diagnosis for MAFLD was 70%. When the VAI was < 1.25, the accuracy of diagnosing MAFLD was 27% (Fig. 8), suggesting that the VAI has good accuracy in diagnosing MAFLD.

## Discussion

In recent years, with the improvement of living standards, the incidence rate of MAFLD has increased each year and has been developing at younger ages. It has become a hidden threat to human health. Although it is a benign disease, it can progress cirrhosis and even hepatocellular carcinoma with the development of the disease and reduce the survival rate of patients[21]. Therefore, the diagnosis of MAFLD is very important for its treatment and prognosis. A study found that the VAI is closely related to the incidence of MAFLD and can be used as an indicator for screening for MAFLD in the general population, but there was no conclusion about its diagnostic efficacy.

Meta-analysis showed that the Sen, Spe, PLR, NLP, DOR, and AUC of the VAI in the diagnosis of MAFLD were 0.70, 0.67, 2.08, 0.39, 5.81, and 0.79, respectively. It is generally believed that the diagnostic efficacy of the AUC is low at 0.50–0.60, medium at 0.70–0.90, high at > 0.90, with a PLR greater than 10.00 and an NLR less than 1.00, indicating that MAFLD can be diagnosed or excluded. From the above analysis, it was found that the VAI has medium diagnostic efficiency in diagnosing MAFLD, and it cannot diagnose MAFLD very accurately.

Heterogeneity is an inevitable problem in meta-analyses. There was heterogeneity caused by the nonthreshold effect in this analysis. Through subgroup analysis and meta-regression analysis, it was found that the heterogeneity was mainly caused by differences in diagnostic methods. The Sen, Spe, PLR, NLR, and DOR values with disease examination as the diagnostic method were higher than those in the imaging examination subgroup, indicating that the diagnostic efficiency of disease examination is better than that of imaging examination. However, imaging examination is still the main means of clinical diagnosis of MAFLD because of its advantages of being noninvasive and having high accuracy [22]. Regarding the source of the control, the diagnostic odds ratio of a healthy population was higher than that of a non-MAFLD population, indicating that the VAI can more accurately diagnose MAFLD when compared with a healthy population. Regarding race, the diagnostic odds ratio for European populations was higher than that for Asian populations, indicating that the VAI can more accurately diagnose MAFLD in European populations. The VAI is a model that is based on clinical data from 315 Italian healthy subjects collected by Marco et al. [6] and was established by regression analysis. There are significant differences in body fat distribution between European populations and Asian populations [23]. This evidence can explain the differences in the diagnostic efficacy of the VAI with different control sources and different races.

In addition, the Fagan diagram shows that when the prior probability is 50%, the posterior probabilities of VAI > 2.33 and VAI < 1.25 are 70% and 27%, respectively. That is, it is assumed that the probability of a diagnosis of MAFLD is 50% according to symptoms and the personal opinions of doctors. After VAI measurement, if the VAI is > 2.33, the accuracy of diagnosis of MAFLD increases to 70%. In contrast, if the VAI is < 1.25, the accuracy of the diagnosis of MAFLD is 20%. Therefore, the VAI-based diagnosis of MAFLD has good clinical application prospects.

### Comparisons with other studies and what the current work adds to existing knowledge

This meta-analysis confirmed for the first time that the VAI can be used as a reliable index for the diagnosis of MAFLD. Patients with a VAI > 2.33 should be highly suspected of having MAFLD, and further examination and diagnosis are recommended.

## Strengths and limitations

This study has the following advantages: (1) All the included studies adopted strict inclusion criteria, and the overall score was high. (2) Subgroup analysis and meta-regression analysis were used to further evaluate whether the analysis results were stable and reliable. (3) The Fagan diagram shows that the VAI has good clinical value.

However, there are still some limitations of this study, considering the limitations of meta-analyses: (1) The failure to obtain unpublished studies may have caused publication bias. (2) Only English documents were included in the study, but Chinese, Japanese, German and other documents were not included, which affects the comprehensiveness of the data. (3) The fact that the critical value of the VAI used for MAFLD diagnosis was not consistent and was affected by the use of different MAFLD diagnostic criteria may have impacted the final result.

## Conclusions

In conclusion, the VAI is an independent predictor for the diagnosis of MAFLD and is feasible for clinical applications. VAI > 2.33 suggests that patients have a high probability of having MAFLD. This work will help screen patients for MAFLD with simple measurement indicators, which will provide evidence for further confirmation of MAFLD and improve the diagnosis rate. Furthermore, this study suggested that targeting visceral adipose tissue in MAFLD patients may be another approach for treatment in the future.

## Supplementary information

**Additional file 1**

## Data Availability

The datasets used and/or analyzed during the current study are available from the corresponding author on reasonable request.

## Abbreviations

VAI: Visceral adiposity index
MAFLD: Metabolic-associated fatty liver disease
TP: True positive value
FP: False positive value
FN: False negative value
TN: True negative value
Sen: Sensitivity
Spe: Specificity
PLR: Positive likelihood ratio
NLR: Negative likelihood ratio
DOR: Diagnostic odds ratio
SROC: Subject operating characteristic curve
AUC: Area under the curve
NOS: Newcastle–Ottawa scale
