# Supplementary material for: Diagnostic accuracy of the visceral adiposity index in patients with metabolic-associated fatty liver disease: a meta-analysis
Source: Lipids Health Dis. 2022 Mar 6;21:28. doi: 10.1186/s12944-022-01636-8 (PMC8898453; doi:10.1186/s12944-022-01636-8)
Supplement: Supplementary file 1 — Additional file 1 [file 12944_2022_1636_MOESM1_ESM.doc]

**Additional file 1 – Search strategy developed for the meta-analysis**

**Medical Databases ---** **Cochrane Library** (Publications until September 1, 2021)

# 1 (“Nonalcoholic Steatohepatitis” [All Text] OR “c nonalcoholic fatty liver disease” [All Text]) OR “metabolic associated fatty liver disease” [All Text] OR “NASH” [All Text] OR “NAFLD” [All Text] OR “MAFLD” [All Text])

# 2 (“Visceral adiposity index” [All Text] OR “VAI” [All Text])

# 3 (#1) AND (#2).

**Medical Databases---Pubmed** (Publications until September 1, 2021)

# 1 (“Nonalcoholic Steatohepatitis” [MeSH Terms] OR “c nonalcoholic fatty liver disease” [MeSH Terms]) OR “metabolic associated fatty liver disease” [MeSH Terms] OR “NASH” [MeSH Terms] OR “NAFLD” [MeSH Terms] OR “MAFLD” [MeSH Terms])

# 2 (“Visceral adiposity index” [MeSH Terms] OR “VAI” [MeSH Terms])

# 3 (#1) AND (#2).

**Medical Databases--- Embase** (Publications until September 1, 2021)

# 1 (“Nonalcoholic Steatohepatitis” [All Fields] OR “c nonalcoholic fatty liver disease” [All Fields]) OR “metabolic associated fatty liver disease” [All Fields] OR “NASH” [All Fields] OR “NAFLD” [All Fields] OR “MAFLD” [All Fields])

# 2 (“Visceral adiposity index” [All Fields] OR “VAI” [All Fields])

# 3 (#1) AND (#2).
